# Protein kinase C activity modulates nuclear Lamin A/C dynamics in HeLa cells

**DOI:** 10.1038/s41598-024-57043-9

**Published:** 2024-03-16

**Authors:** Chase C. Wesley, Dallin V. North, Daniel L. Levy

**Affiliations:** Department of Molecular Biology, University of Wyoming, 1000 E. University Avenue, Laramie, WY 82071 USA

**Keywords:** Nuclear organization, Nucleus

## Abstract

The nuclear lamina serves important functions in the nucleus, providing structural support to the nuclear envelope and contributing to chromatin organization. The primary proteins that constitute the lamina are nuclear lamins whose functions are impacted by post-translational modifications, including phosphorylation by protein kinase C (PKC). While PKC-mediated lamin phosphorylation is important for nuclear envelope breakdown during mitosis, less is known about interphase roles for PKC in regulating nuclear structure. Here we show that overexpression of PKC ß, but not PKC α, increases the Lamin A/C mobile fraction in the nuclear envelope in HeLa cells without changing the overall structure of Lamin A/C and Lamin B1 within the nuclear lamina. Conversely, knockdown of PKC ß, but not PKC α, reduces the Lamin A/C mobile fraction. Thus, we demonstrate an isoform-specific role for PKC in regulating interphase Lamin A/C dynamics outside of mitosis.

## Introduction

The nuclear lamina is a protein meshwork found on the innermost side of the nuclear envelope^1,2^. Canonically providing structural support for the nucleus, the nuclear lamina also plays roles in chromatin organization and RNA splicing^3–10^. The nuclear lamina consists mainly of a set of proteins termed nuclear lamins. There are four main lamin proteins in human cells: Lamin B1, Lamin B2, Lamin A, and Lamin C, which is a shortened version of Lamin A created via alternative splicing^10–16^. Other, less common variants of these proteins are restricted to certain cell types and are also created as a result of alternative splicing^17,18^. Lamin mutations are associated with a variety of genetic diseases, collectively termed laminopathies. Of these conditions, laminopathies associated with Lamin A/C mutations are most common and range from muscular dystrophies and lipodystrophies to the premature aging syndrome Hutchinson-Gilford Progeria^19–23^. Thus, nuclear lamins play critical roles in cell and nuclear function.

Nuclear lamins undergo numerous post-translational modifications that alter their function, including acetylation, succinylation, methylation, and ubiquitination, with phosphorylation being the most common^24–30^. Each lamin protein contains dozens of phosphorylation sites that can be modified by a number of kinases^31–33^. Lamin phosphorylation by CDK1 and protein kinase C (PKC) plays a well-established role in nuclear envelope breakdown during mitosis^34–36^. However, other roles for lamin phosphorylation are known. For example, phosphorylation of Lamin B3 by conventional PKC reduces the size of *Xenopus* nuclei and contributes to developmental scaling of nuclear size during early *Xenopus* embryogenesis^37^. PKC-mediated phosphorylation of Lamin A/C also affects nuclear size in mammalian cells^38^. Open questions from this latter study that we address here are whether PKC activity influences Lamin A/C dynamics and whether any effects are PKC isoform-specific.

## Results

**Figure 1:**
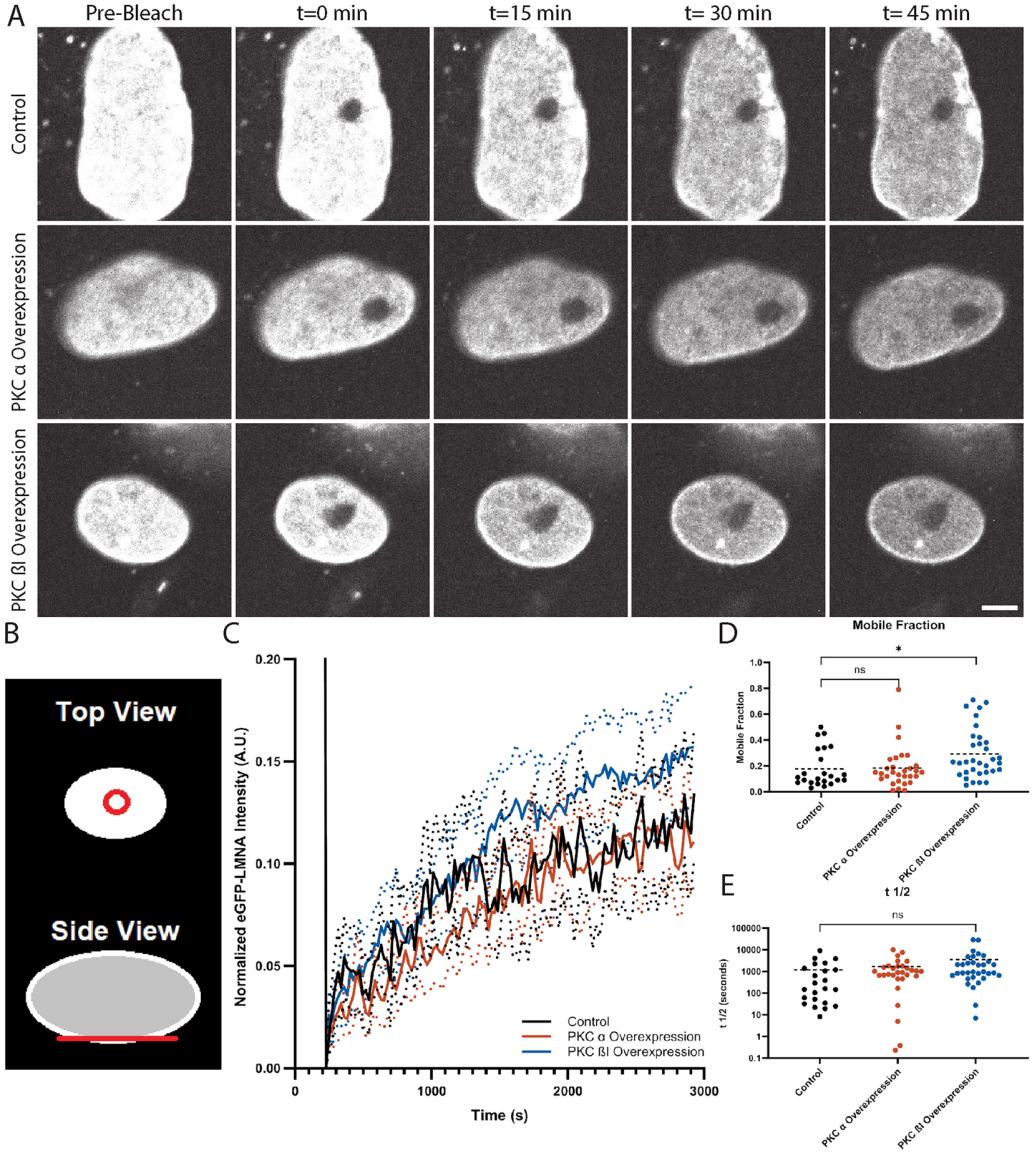
PKC βI overexpression increases the Lamin A/C mobile fraction. Genome-edited eGFP-LMNA HeLa cells were transfected with plasmids expressing mCherry (Control), mCherry-PKC α-dNPS (PKC α overexpression), or mCherry-PKC βI-dNPS (PKC β1 overexpression). Imaging of eGFP-LMNA was performed. A circular region of eGFP-LMNA within the NE closest to the coverslip was photobleached (see B) and FRAP time lapses were acquired. 10 prebleach images and 121 postbleach images were acquired at regular time intervals for each time lapse. The total postbleach acquisition time was 45 min. (**A**) Images of eGFP-LMNA from representative FRAP timelapses are shown. Scale bar: 5 µm. (**B**) Diagram showing how a circular region of eGFP-LMNA within the NE closest to the coverslip was photobleached (red lines). (**C**) Average fluorescence recovery curves based on full scale normalized data (see “Materials and methods”). Solid lines are normalized average fluorescence. Dashed lines are 95% confidence intervals. (**D**) Mobile fraction values were calculated as described in Materials and Methods. (**E**) t_1/2_ values were calculated as described in Materials and Methods. Y-axis shows base 10 logarithmic scale. On scatter plots, dashed lines represent means. Data were acquired for 23 nuclei from the mCherry control group, 30 nuclei from the mCherry-PKC α-dNPS group, and 33 nuclei from the mCherry-PKC βI-dNPS group based on three independent trials for each group. One-way ANOVA with Dunnett’s multiple comparisons statistical tests were performed, with significance relative to the mCherry control shown. ns, not significant; *p < 0.05.

To investigate Lamin A/C dynamics, we employed fluorescence recovery after photobleaching (FRAP). First, we used CRISPR to generate a genome-edited HeLa cell line with an eGFP tag fused to the N-terminus of Lamin A/C (Fig. S1A). One *LMNA* allele was tagged, and a homogeneous clonal cell line was isolated (see “Methods”). We validated this new cell line by showing that Lamin A/C knockdown reduced the intensity of the eGFP signal by microscopy (Fig. S1A,B) and immunoblotting (Fig. S1C,D). Immunoblotting also established that the tagging was monoallelic and did not significantly affect total Lamin A/C protein levels (Fig. S1C,D). Next, we performed transient transfections with plasmids expressing constitutively active PKC isoforms fused to mCherry or mCherry alone as a control. These constitutively active PKC variants lack the N-terminal pseudosubstrate domain and have been shown to exhibit kinase activity in cultured cells^39^. We validated ectopic PKC expression by both immunofluorescence and immunoblotting (Fig. S2). To perform FRAP, we focused on the portion of the nuclear envelope closest to the coverslip and bleached a circular region of eGFP-LMNA signal (Fig. 1A,B). Recovery of eGFP-LMNA signal into this region was quantified over time and analyzed as described in Materials and Methods to generate the recovery half-time (t_1/2_) and mobile fraction. The half-time reports on how quickly Lamin A/C moves within the nuclear envelope, and the mobile fraction indicates how much of the Lamin A/C protein is mobile.

Cells transfected with constitutively active PKC ßI exhibited an increased eGFP-LMNA mobile fraction compared to control while PKC α expression had no effect (Fig. 1C,D). These data indicate that PKC ßI activity specifically increases the amount of Lamin A/C that dynamically associates with the nuclear envelope. Interestingly, the eGFP-LMNA recovery rate was not affected by expression of either PKC isoform (Fig. 1E). This suggests that PKC ß modulates the proportion of Lamin A/C that is mobile within the nuclear envelope but not necessarily how rapidly the Lamin A/C moves or associates.

**Figure 2:**
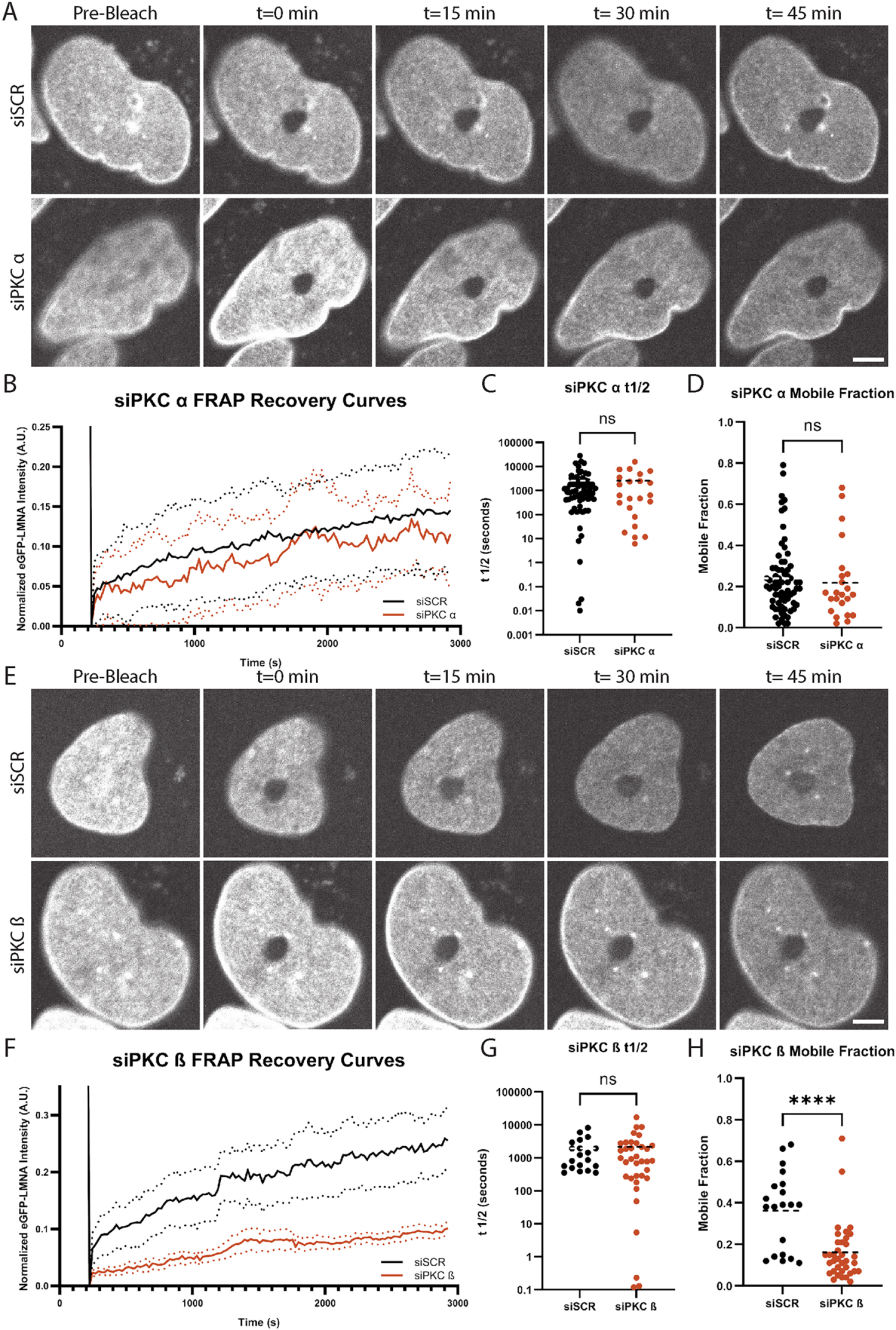
PKC β knockdown decreases the Lamin A/C mobile fraction. eGFP-LMNA HeLa cells were transfected with siRNAs targeting negative control (siSCR), PKC α (siPKC α), or PKC β (siPKC β). FRAP experiments were performed and analyzed as in Fig. 1. (**A**–**D**) PKC α knockdown experiments. (**E**–**H**) PKC β knockdown experiments. (**A**,**E**) Images from representative timelapses are shown. Scale bars: 5 µm. (**B**,**F**) Average fluorescence recovery curves based on full scale normalized data are shown. Solid lines are normalized average fluorescence. Dashed lines are 95% confidence intervals. (**C**,**G**) t_1/2_ values are shown. (**D**,**H**) Mobile fraction values are shown. On scatter plots, dashed lines represent means. For (**A**–**D**), data were acquired for 69 nuclei from the siSCR negative control group and 23 nuclei from the siPKC α group based on at least two independent trials. For (**E**–**H**), data were acquired for 20 nuclei from the siSCR negative control group and 36 nuclei from the siPKC β group based on three independent trials for each group. Unpaired, two-tailed t tests were performed. ns, not significant; ****p < 0.0001.

Our overexpression data suggested that PKC ß is a positive regulator of Lamin A/C dynamics. To further explore this hypothesis, we tested PKC knockdown. We transfected our eGFP-LMNA HeLa cells with siRNA targeting either PKC α, PKC ß, or a scrambled control. We confirmed PKC α knockdown by immunofluorescence and immunoblotting (Fig. S3), and our previous study established effective PKC ß knockdown in HeLa cells^38^. While PKC α knockdown had little effect on Lamin A/C dynamics (Fig. 2A–D), PKC ß knockdown led to a significant reduction in eGFP-LMNA recovery corresponding to a much smaller eGFP-LMNA mobile fraction (Fig. 2E–H). Consistent with our overexpression studies, these data show that Lamin A/C dynamics are sensitive to levels of PKC ß but not PKC α.

**Figure 3:**
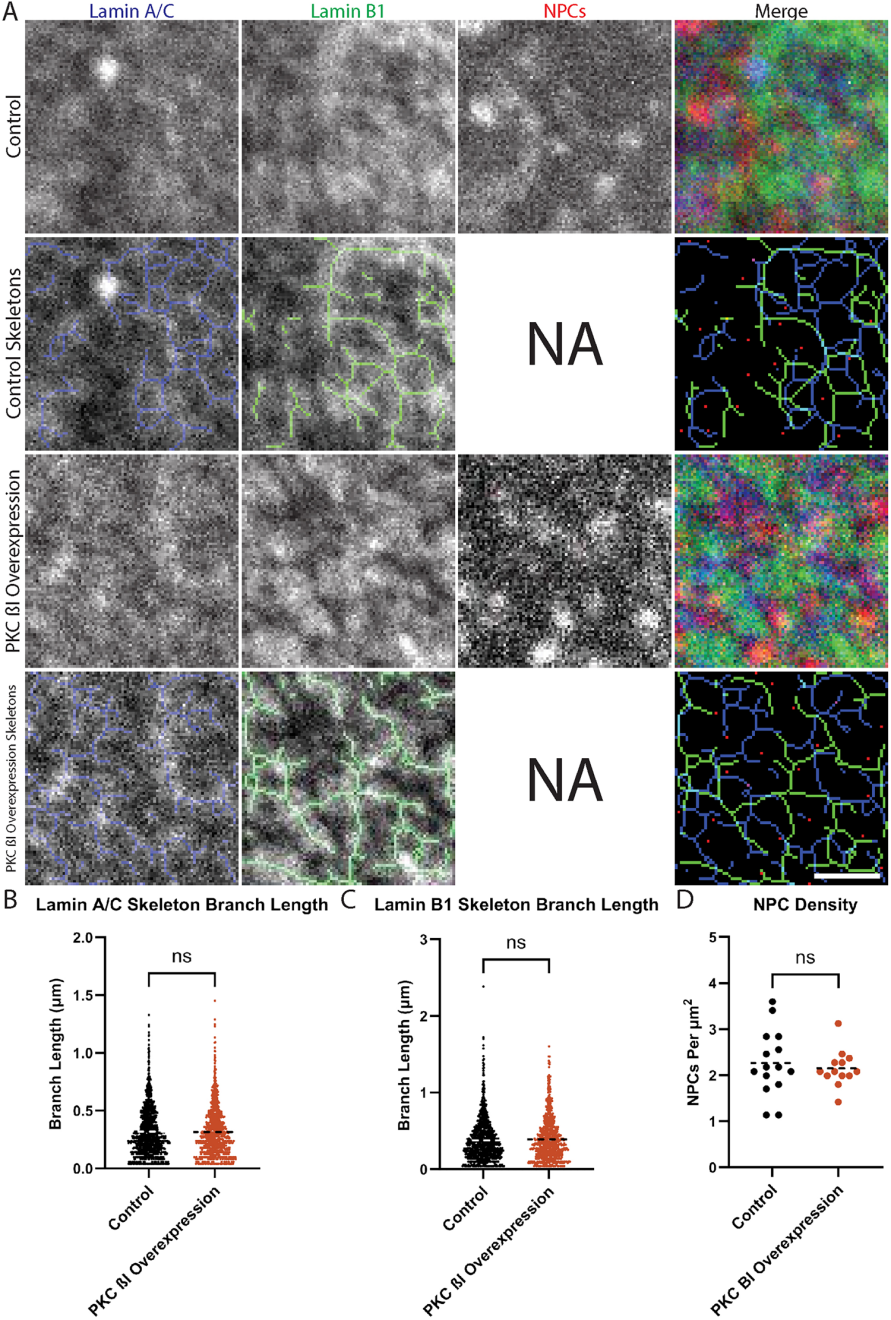
PKC βI overexpression does not alter overall nuclear lamina organization or NPC density. (**A**) eGFP-LMNA HeLa cells were transfected with plasmids expressing mCherry (Control) or mCherry-PKC βI-dNPS (PKC β1 overexpression). Cells were stained with α-Lamin B1 and α-NPC antibodies and superresolution imaging was performed. Images were subjected to skeletonization as described in Materials and Methods. Representative images are shown. Scale bar: 1 µm. NA indicates skeletonization not applicable for NPC staining. (**B**) Lamin A/C branch lengths were quantified. (**C**) Lamin B1 branch lengths were quantified. (**D**) NPC densities were quantified. On scatter plots, dashed lines represent means. For (**B**), 1419 control and 1222 PKC β overexpression Lamin A/C branches were quantified. For (**C**), 1040 control and 853 PKC β overexpression Lamin B1 branches were quantified. For (**D**), 15 control nuclei and 13 PKC β overexpression nuclei were quantified. All data were collected from three biological replicates. Unpaired, two-tailed t tests were performed. ns, not significant.

Given that PKC ß influences Lamin A/C dynamics, we next sought to investigate if PKC ß affects the overall organization of the nuclear lamina and positioning of nuclear pore complexes (NPCs), given that NPCs interact with the lamina^40,41^ and can affect nuclear size^42–48^. To analyze lamin and NPC organization, our genome-edited eGFP-LMNA HeLa cells were transfected with plasmids expressing constitutively active PKC ßI or mCherry as a control, subjected to immunofluorescence for Lamin B1 and NPCs, and imaged using superresolution microscopy (Fig. 3A). For quantitative analysis, images of Lamin A/C and Lamin B1 were skeletonized and lamin branch lengths were measured^49^. Based on this analysis, we found that Lamin A/C and Lamin B1 branch lengths were unchanged by PKC ßI overexpression (Fig. 3B,C), and NPC density was also unaltered (Fig. 3D). Taken together, these data indicate that PKC ß activity modulates Lamin A/C dynamics without leading to large-scale rearrangements of the nuclear lamina and NPCs.

## Discussion

Our data show that PKC ß activity can impact the dynamics of Lamin A/C. Increasing and decreasing PKC ß levels conversely affected the Lamin A/C mobile fraction, suggesting that PKC activity contributes to Lamin A/C dynamics under normal growth conditions. Interestingly, altering PKC α activity did not affect Lamin A/C dynamics, indicating that the effect is isoform-specific and possibly related to specific interphase phosphorylation sites on Lamin A/C. Identifying the PKC ß phosphorylation sites in Lamin A/C that mediate its dynamic association with the nuclear lamina is a topic for future investigation. Observed changes in Lamin A/C dynamics only impacted the mobile fraction, indicating that PKC activity affects the amount of Lamin A/C that dynamically associates with the nuclear lamina but not its rate of association. It is important to note that we measured Lamin A/C dynamics in interphase nuclei after post-mitotic nuclear envelope assembly. It is possible that PKC activity affects the Lamin A/C association rate with the nuclear lamina at the beginning of the G1 phase of the cell cycle during nuclear envelope reformation, but that PKC activity does not affect the association rate later in interphase when the lamins are more stably associated with the nuclear lamina. An effect on mobile fraction may be more physiologically relevant than recovery rate, as the amount of Lamin A/C that is stably versus transiently associated with the nuclear envelope could impact chromatin organization and structural properties of the nucleus.

It is interesting to note that, in spite of PKC ß-mediated changes in Lamin A/C dynamics, PKC ß activity did not affect the gross structure of the nuclear lamina nor NPC organization. It is possible that there are more subtle changes in these structures that we could not detect by light microscopy and higher resolution techniques can be used to explore this issue further. Nonetheless, our data suggest that the overall organization of the nuclear envelope and lamina are largely maintained even when Lamin A/C dynamics are altered. This observation is consistent with the idea that multiple redundant interactions reinforce the structure of the nuclear lamina. While it is formally possible that observed PKC-mediated changes in interphase Lamin A/C dynamics could result from altered disassembly and/or assembly of the nuclear lamina in the previous cell cycle, this seems unlikely given that overall nuclear lamina organization and NPC density were unaffected (Fig. 3) and no significant changes in cell proliferation were observed.

A previous study reported that PKC ß acts as a negative regulator of nuclear size in cultured mammalian cells^38^. It is tempting to speculate that PKC-mediated changes in nuclear size are related to altered Lamin A/C dynamics. For example, an increase in Lamin A/C dynamics could alter the mechanical properties of the nuclear envelope, perhaps resulting in a softer nucleus with reduced nuclear size. Another possibility is that Lamin A/C dynamics influence the recruitment of lamin-associated proteins that, in turn, modulate nuclear size. In any case, our study demonstrates a role for PKC ß in the regulation of Lamin A/C dynamics and future work will explore the significance of this regulation for nuclear and cell function.

## Materials and methods

### Antibodies

Portions of this section were published previously^50^. The following antibodies were used at the indicated concentrations: mouse anti-Lamin A/C used at 1:1000 for immunofluorescence (Santa Cruz Biotechnology; sc-376248), rabbit anti-Lamin A/C used at 1:1000 for Western Blotting (Cell Signaling Technology, 2032 T), rabbit anti-PKC β used at 1:500 for PKC ß overexpression immunofluorescence (GTX 113252), rabbit anti-PKC β used at 1:1000 for western blotting (Proteintech, 12919-1-AP), mouse anti-PKC α used at 1:50 for immunofluorescence and 1:200 for Western Blotting (Santa Cruz Biotechnology, sc-8393), rabbit anti–lamin B1 used at 0.35 µg/ml for immunofluorescence (Abcam; ab16048), mouse anti–nuclear pore complex used at 1:1000 for immunofluorescence (mAb414; Biolegend; 902901), mouse anti–α-tubulin (DM1A) used at 1:200 for Western blotting (Santa Cruz Biotechnology; sc-32293), rabbit anti–β-actin used at 1:1000 for Western blotting (RevMAb Biosciences; 31-1013-00), goat anti-rabbit IgG H&L Alexa Fluor 405 used at 1:200 for immunofluorescence (Abcam; ab175652), donkey anti-mouse IgG H + L Alexa Fluor™ 568 (ThermoFisher; A10037) used at 1:1000 for immunofluorescence, donkey anti-mouse IgG H + L Alexa Fluor™ 647 used at 1:1000 for immunofluorescence (ThermoFisher; A-31571), goat anti-rabbit 647 IgG (Heavy chain) (ThermoFisher; A27040) used at 1:1000 for immunofluorescence, IRDye 680RD goat anti-mouse IgG used at 1:15,000 for Western blotting (Li-Cor; 926-68070), and IRDye 800RD anti-rabbit IgG used at 1:10,000 for Western blotting (Li-Cor; 926-32211).

### HeLa cell culture

HeLa cells were cultured at 37 °C with 5% CO_2_ in Eagle’s minimum essential medium supplemented with 10% fetal bovine serum and 50 IU/mL penicillin and streptomycin. For transfections, cells were grown in 3.5 cm dishes, 6-well dishes, µ-Slide 2 Well Dishes (Ibidi; 80286), or µ-Slide 8 Well Dishes (Ibidi; 80821). For immunofluorescence experiments, acid-washed 22 × 22 mm square no. 1½ glass coverslips (Corning; 2850–22) were added to 3.5 cm dishes or 6-cell dishes prior to seeding with HeLa cells. We note that HeLa cell nuclear morphology is heterogeneous^38,51–53^.

### FRAP experiments

Cells were cultured in µ-Slide 2 Well dishes (Ibidi; 80286) or in µ-Slide 8 Well Dishes (Ibidi; 80821). Photobleaching of eGFP-Lamin A/C at the NE was accomplished with ten pulses of a 405-nm laser set at 100% power in a circular region 0.9 µm in diameter. Time lapses were acquired with 10 prebleach time points and 121 postbleach time points taken at 22.5 s intervals for a 45-min postbleach period. FRAP time lapses were analyzed using ImageJ Fiji software recording the mean fluorescence of the bleached region, the whole nucleus, and a background region for each time point^54^. This normalization is designed to adjust for background signal, differences in starting intensity of the photobleached region, and differences in the total fluorescence over the course of the time lapse. The data from this analysis were processed using easyFRAP software^55^. This software first subtracts the background intensity from the intensity of the nucleus and the photobleached region at every time point, and then calculates the average fluorescence intensity of the nucleus and the average fluorescence intensity of the photobleached region before photobleaching. The normalization is then calculated at each time point (t) with the following equation:

$$F\left(t\right)=\frac{{N}_{pre}}{N(t)}*\frac{B\left(t\right)}{{B}_{pre}},$$

where F(t) is the normalized fluorescence at each time point (i.e. the double normalized fluorescence), N_pre_ is the average intensity of the nucleus before the photobleach, N(t) is the intensity of the nucleus at each time point, B(t) is the average intensity of the bleached region at each time point, and B_pre_ is the average intensity of the bleached region before the photobleach. Furthermore, the data are then normalized using a full scale normalization. For this type of normalization, the following equation is used:

$${F(t)}_{\begin{array}{c}fullscale\\ norm\end{array}}=\frac{{F(t)}_{\begin{array}{c}double\\ norm\end{array}}-{F({t}_{postbleach})}_{double norm}}{1-{F({t}_{postbleach})}_{double norm}},$$

where F(t)_fullscale norm_ is the full scale normalized fluorescence at each time point, F(t)_double norm_ is the double normalized fluorescence at each time point, and F(t_postbleach_)_double norm_ is the double normalized fluorescence of the data immediately after the photobleach. This normalizes the data so that the fluorescence intensity starts at 0 just after the photobleach. For each curve, t_1/2_ (the amount of time for half of the fluorescence recovery to occur) and mobile fraction (the total amount of fluorescence recovery to occur as a fraction of the total possible recovery) were calculated^55^. Curves with mobile fractions of 0 or ≥ 0.8 were removed from each dataset as outliers.

### Microscopy

Portions of this section were published previously^50^. Confocal microscopy images for FRAP experiments were obtained using an Olympus IX81 microscope stand equipped with a Yokogawa CSU-X1 spinning-disk confocal head, a five-line LMM5 laser launch (Spectral Applied Research), and Hamamatsu ORCA-Flash4.0 C114400 digital CMOS camera. Samples were maintained at 37 °C and 5% CO_2_ with a stage-top incubator (Tokai Hit; INUBG2A-ZILCS). An Olympus UPlanSApo 60 × /1.20 water immersion objective was used. An iLas2 system was used to control FRAP experiments. Confocal superresolution microscopy images were acquired using an Olympus IX83 inverted confocal microscope equipped with an IXplore SpinSR microscope system, a Coherent OBIS LX 405 nm, 50 mW laser system (SKU 1284370), a Coherent OBIS LS 488 nm, 100-mW laser system (SKU 1226420), a Coherent OBIS LS 561 nm, 100-mW laser system (SKU 1253302), a Coherent OBIS LX 640 nm, 100-mW laser system (SKU1178790), a Yokogawa CSU-W1 SoRa confocal scanner unit, ORCA-Fusion C14440 digital cameras, and an Olympus U-RTCE real-time controller. Image acquisition was controlled using a motorized Olympus stage, a Marzhauser Wetzlar TANGO Desktop controller, and CellSens software. The objective used was an Olympus UPlanSApo 100 × (NA 1.35; silicon oil) with a 3.2 × magnification changer for superresolution imaging. Nuclei were analyzed as z-stacks with a 0.2-µm step size for most experiments including superresolution imaging for Lamin A/C and Lamin B1 skeleton analysis and NPCs location analysis. Images for measuring fluorescence intensity in an individual experiment were acquired using the same exposure time.

### Immunofluorescence

Portions of this section were published previously^50^. For immunofluorescence staining, cells were grown on acid-washed 22 × 22 mm square no. 1½ glass coverslips (Corning; 2850–22). Cells on coverslips were washed three times in phosphate-buffered saline (PBS) for 5 min per wash, then fixed using 4% paraformaldehyde for 15 min. Cells on coverslips were then washed three times in PBS for 5 min per wash and permeabilized with 0.1% Triton X-100 for 10 min. After three more 5-min PBS washes, cells were blocked in 10% goat serum (Sigma; G9023) and 0.3 M glycine in PBS for 1 h. Three 5-min washes in PBS were repeated and cells were stained overnight in primary antibody solution (2% goat serum in PBS with appropriate primary antibodies). After three more 5-min PBS washes, cells were incubated with secondary antibody solution (2% goat serum in PBS with appropriate secondary antibodies) for 75 min. Hoechst 33342 stain (Sigma; 14533) was added to the secondary antibody solution at a concentration of 10 µg/mL in some assays to image DNA. Coverslips were then washed three times in PBS for 5 min per wash, briefly washed twice with dH_2_O, mounted on microscope slides using VECTASHIELD antifade mounting medium (Vector Laboratories; H-1000), and sealed with nail polish.

### Image analysis

To measure the intensity of fluorescence signals at the nucleus (i.e. Lamin A/C, eGFP-Lamin A/C, and Hoechst signals), Hoechst images of nuclei were viewed in ImageJ and thresholded to select nuclei. Nuclei were separated manually if necessary. These selections were then used to measure fluorescence signal intensity. A region with no signal was selected and used to background subtract all measured fluorescence signals. To measure the fluorescence signal for PKC α and PKC β, whole cells were thresholded after a gaussian blur was applied with a sigma of 0.65 µm. Cells were separated manually if necessary. These selections were used to measure the fluorescence signal of each cell. As with the nuclear measurements, a section with no signal was selected and used to background subtract all measured fluorescence signals.

To measure Lamin A/C branch length, Lamin B1 branch length, and NPC position, eGFP-LMNA HeLa nuclei were immunostained for Lamin B1 and NPCs and imaged by superresolution microscopy on the nucleus surface. A Gaussian blur with a sigma of 0.041 µm was applied to the images of the lamina before thresholding to prevent detection of extraneous branches. A 10.6 µm^2^ square region was selected at the lamina of each nucleus in ImageJ. The selected region was then Li thresholded, made binary, skeletonized, and measured for branch length as described in Ref.^49^.

### Western blots

Whole cell lysates were prepared using RIPA buffer and incubated on ice for 30 min, after which lysates were centrifuged to pellet insoluble cellular debris and snap-frozen in liquid nitrogen. For normalization, lysate protein concentrations were measured using a EZQ Protein Quantitation Kit (Thermofisher; R33200). Lysate proteins were separated on 10% SDS-PAGE gels. Proteins were transferred to PVDF by semidry transfer. Membranes were then blocked in 5% milk in PBS, probed with primary antibodies overnight, and stained with Li-Cor Odyssey secondary antibodies (see Antibodies section). Blots were then scanned using a Li-Cor Odyssey CLx imager. Band intensities were quantified using ImageJ software and normalized for background signal. To quantify total protein, blots were stained with Ponceau (0.1% wt/vol Ponceau in 5% acetic acid), destained in 5% acetic acid, rinsed in distilled water, imaged on a BioRad ChemiDoc MP imager, analyzed using ImageJ software, and normalized for background signal^54^. Full-length western blots are shown in Fig. S4.

### Transfections

DNA transfections were carried out using 3.75 µL Lipofectamine 3000, 5 µL P3000™ reagent, and 2500 ng DNA per 3.5 cm dish, as per the manufacturer’s instructions (Thermofisher, L3000008). RNA transfections were carried out using 7.5 µL Lipofectamine™ RNAi Max (Thermofisher, 13778075) per 3.5 cm dish as per the manufacturer’s instructions. For siPKC α transfections and their corresponding controls, 15 pmol RNA were used per 3.5 cm dish. For siPKC ß transfections and their corresponding controls, 25 pmol RNA were used per 3.5 cm dish. RNA transfections also used 12.5 pmol BLOCK-iT™ Alexa Fluor™ Red Fluorescent Control (ThermoFisher, 14750100) per dish in addition to the siRNAs added. All assays were conducted 48 h post-transfection.

For FRAP experiments, transfections were performed in µ-Slide 2 Well Dishes (Ibidi; 80286) or in µ-Slide 8 Well Dishes (Ibidi; 80821). Transfections were performed the same way except for µ-Slide 2 Well Dishes we used 10.9 pmol siRNA for siPKC β transfections and controls, while 6.5 pmol siRNA was used for siPKC α transfections and controls. In both cases, 3.27µL Lipofectamine™ RNAi Max (Thermofisher, 13778075) and 5.5 pmol BLOCK-iT™ Alexa Fluor™ Red Fluorescent Control was used. For µ-Slide 8 Well Dishes, we used 2.1 pmol siRNA for siPKC β transfections and controls, while 1.3 pmol siRNA was used for siPKC α transfections and controls. In both cases, 0.638µL Lipofectamine™ RNAi Max (Thermofisher, 13778075) and 1.1 pmol BLOCK-iT™ Alexa Fluor™ Red Fluorescent Control was used. For DNA transfections, 1.636µL Lipofectamine 3000, 2.18µL P3000™ reagent, and 1.1 µg DNA was used for transfections in µ-Slide 2 Well Dishes and 0.319µL Lipofectamine 3000 (Thermofisher, L3000008), 0.425µL P3000™ reagent (Thermofisher, L3000008), and 0.2 µg DNA was used for transfections in µ-Slide 8 Well Dishes.

### RNAi

For siRNA transfections, the following siRNAs were used: PKC α (SASI_Hs01_00018815; Sigma-Aldrich), PKC β (SASI_Hs01_00071183; Sigma-Aldrich), LMNA (SASI_Hs02_00367643; Sigma-Aldrich), and universal negative control (SIC001; Sigma-Aldrich).

### Molecular cloning

The pEmCherry-C2 plasmid is a modified version of pEGFP-C2 provided by Anne Schlaitz (Heidelberg University) and was used to create pEmCherry-PKC α-dNPS and pEmCherry-PKC βI-dNPS plasmids. This was accomplished by PCR amplifying PKC α-dNPS from a PKC α-dNPS plasmid (Addgene, 21233) and PKC βI-dNPS from a PKC βI-dNPS plasmid (Addgene, 16379), restriction digestion with XhoI (New England BioLabs Inc., R0146S) and XmaI (New England BioLabs Inc., R0180S), and cloning them into the pEmCherry-C2 plasmid. For the PCR amplification, the following primers were used:

PKC α/ßI Forward Primer: 5’- CACCATCTCGAGCAACGTGCACGAGGTG -3’.

PKC α Reverse Primer: 5’- AGGATACCCGGGTACTGCACTCTGTAAGATGGG -3’.

PKC ßI Reverse Primer: 5’-AGGATACCCGGGCACATTAATGACAAACTCTGGG -3’.

To create the eGFP-LMNA repair construct for genome editing, a 1408 base pair region around the translation start site of the LMNA gene was PCR amplified from a human genomic template (human male DNA, Applied Biosystems, part #360,486) with the following primers:

LMNA Homology Region Forward Primer:

5’-AAGGAACTCGAGTTCCAGAACTTTGCTCCCCCCAGGGAACCCAGG -3’.

LMNA Homology Region Reverse Primer:

5’-AGCCCCGGATCCTCCCTGATACCCCCACCATTCCTTATATCCTCC-3’.

This amplified region was then cloned into a pBlueScript II KS (-) plasmid restriction digested with XhoI (New England BioLabs Inc., R0146S) and BamHI. The eGFP region of the pDL74 plasmid^56^ was then PCR amplified with the following primers:

eGFP Forward Primer: 5’-AACCTGCCGGCCATGGTGAGCAAGGGC -3’.

eGFP Reverse Primer: 5’-GACGGGGTCTCCATGGAATTCCCTCCACCC -3’.

This region was then cloned into the LMNA translation start site in the pBlueScript II KS (−) vector after digestion with NcoI-HF (New England BioLabs, R3193S). For genome editing, the LMNA and eGFP regions of this plasmid were PCR amplified using the same primers used to amplify the region around the start site of the LMNA gene. This product was purified using a QIAquick Gel Extraction Kit (Qiagen, Cat. No. 28704) and used as the homology repair domain (HDR).

### Genome editing

HeLa cells were genome edited using the protocol detailed in MilliporeSigma^57^ with the protocol adjusted to use 60 pmol Cas9 (Sigma-Aldrich, CAS9PROT-250UG) and 120 pmol gRNA (mC* mC* mG* rC rU rG rG rG rA rC rG rG rG rG rU rC rU rC rC rA rG rU rU rU rU rA rG rA rG rC rU rA rG rA rA rA rU rA rG rC rA rA rG rU rU rA rA rA rA rU rA rA rG rG rC rU rA rG rU rC rC rG rU rU rA rU rC rA rA rC rU rU rG rA rA rA rA rA rG rU rG rG rC rA rC rC rG rA rG rU rC rG rG rU rG rC mU* mU* mU* rU, Integrated DNA Technologies, Alt-R CRISPR-Cas9 sgRNA). Both of these were combined, incubated for 5 min, and added to a mix of 82 µL nucleofection solution and 18 µL nucleofector supplement from a SE Cell Line 4D-Nucleofector X Kit L (Lonza; V4XC-1012). To this nucleofection solution, 2.56 pmol HDR was added. Subsequently, this combined solution was used to resuspend 2.5 × 10^5^ HeLa cells. The cell-containing solution was then added to a 100 µL Nucleocuvette™ and the nucleofection was carried out using the “HeLa” nucleofection setting on a Lonza 4D-Nucleofector™. After transfection, HeLa cells were grown under standard culture conditions until cells with strong GFP fluorescence were sorted out using a BD FACSMelody™ Cell Sorter. Untransfected HeLa cells were used as negative control for this process. Sorted cells were then cultured at low confluency on a 10 cm petri dish and individual colonies of cells were isolated using cloning cylinders. These colonies were then trypsinized, seeded into individual wells of µ-Slide 8 Well Dishes, and imaged to verify the presence of eGFP. Cells were then used under normal culture conditions and one clone was selected to be verified for the correct insertion of eGFP in the LMNA allele (see Fig. S1). As such all of our experiments were performed with a cell line purified to homogeneity. In the cell line we selected, only one LMNA allele was modified. The GFP-tagged LMNA is expressed at a lower level than wild-type LMNA because HeLa cells are hypertriploid^58,59^ meaning the number of wild-type alleles outnumbers the single GFP-tagged allele and/or because the tagging itself reduces expression, as was previously noted for CRISPR-mediated GFP tagging of Lamin B1^50^.

### Supplementary Information

Supplementary Figures.

## Data Availability

All data and materials associated with this study are available upon request from corresponding author Daniel Levy (dlevy1@uwyo.edu).
