# Supplementary material for: Protein kinase C activity modulates nuclear Lamin A/C dynamics in HeLa cells
Source: Sci Rep. 2024 Mar 16;14:6388. doi: 10.1038/s41598-024-57043-9 (PMC10944469; doi:10.1038/s41598-024-57043-9)
Supplement: Supplementary file 1 — Supplementary Figures. [file 41598_2024_57043_MOESM1_ESM.pdf]

Supplemental Materials

Protein kinase C activity modulates nuclear Lamin A/C dynamics in HeLa cells

Chase C. Wesley, Dallin V. North, and Daniel L. Levy*
Department of Molecular Biology, University of Wyoming, Laramie, WY, 82071, USA

*Corresponding author:
Daniel L. Levy
University of Wyoming
Department of Molecular Biology
1000 E. University Avenue
Laramie, WY, 82071
Phone: 307-417-0354
Fax: 307-766-5098
E-mail: dlevy1@uwyo.edu

Running Head: PKC-mediated Lamin A/C dynamics

Supplemental figure legends

Figure S1: Validation of the eGFP-LMNA HeLa cell line. (A-B) Genome-edited eGFP-LMNA HeLa cells were transfected with siRNAs targeting negative control (siSCR) or Lamin A/C (siLMNA). **(A)** Representative images of the GFP signal are shown. Scale bar: 5 μm. **(B)** GFP fluorescence signal intensities were quantified. Data are shown for 1607 siSCR nuclei and 1397 siLMNA nuclei from 3 biological replicates. **(C-D)** Wild-type (WT) and eGFP-LMNA HeLa cells were transfected with siRNAs targeting negative control (siSCR) or Lamin A/C (siLMNA). Cell lysates for three biological replicates were subjected to western blotting with antibodies against Lamin A/C and α-tubulin. **(C)** The western blot is shown. The blue line delineates two separate blots; the top blot was probed for Lamin A/C and the bottom blot was probed for α-tubulin. **(D)** Total Lamin A/C signal was quantified for each lane and normalized to the total protein amount measured from the Ponceau-stained membrane.
On scatter plots, dashed lines represent means. Unpaired, two-tailed t tests were performed. ns, not significant; *, $p < 0.05$; **, $p<0.01$; ****, $p<0.0001$.

Figure S2: Validation of PKC overexpression. Genome-edited eGFP-LMNA HeLa cells were transfected with plasmids expressing mCherry (Control), mCherry-PKC α-dNPS (PKC α overexpression), or mCherry-PKC βI-dNPS (PKC β1 overexpression). **(A)** Immunofluorescence (IF) was performed with an antibody against PKC α. Representative images are shown. Scale bar: 10 μm. **(B)** Based on the PKC α IF performed in (A), total PKC α signal was quantified for 362 mCherry Control cells and 301 mCherry-PKC α-dNPS cells based on three biological replicates. Y-axis shows base 10 logarithmic scale. **(C)** HeLa cell lysates were subjected to Western blotting using antibodies against PKC α and β-actin. The blue line delineates two separate blots; the top blot was probed for PKC α and the bottom blot was probed for β-actin. The total integrated PKC α band intensity signal was quantified and normalized to the total protein signal measured by Ponceau staining. Each lane represents one biological replicate. The normalized total PKC α signal was 0.0335 ± 0.0070 for mCherry control and 0.0788 ± 0.040 for mCherry-PKC α-dNPS. **(D)** IF was performed with an antibody against PKC β. Representative images are shown. Scale bar: 10 μm. **(E)** Based on the

PKC β IF performed in (D), total PKC β signal was quantified for 362 mCherry Control cells and 302 mCherry-PKC βI-dNPS cells based on three biological replicates. Y-axis shows base 10 logarithmic scale. **(F)** HeLa cell lysates were subjected to Western blotting using antibodies against PKC β and α-tubulin. The blue line delineates two separate blots; the top blot was probed for PKC β and the bottom blot was probed for α-tubulin. The total integrated PKC β band intensity signal was quantified and normalized to the total protein signal measured by Ponceau staining. Each lane represents one biological replicate. The normalized total PKC β signal was 0.158 ± 0.058 for mCherry control and 0.249 ± 0.112 for mCherry-PKC βI-dNPS.
On scatter plots, dashed lines represent means. Unpaired, two-tailed t tests were performed. ****, $p<0.0001$.

Figure S3: Validation of PKC α knockdown. Genome-edited eGFP-LMNA HeLa cells were transfected with BLOCK-iT™ Alexa Fluor™ Red Fluorescent Control (Transfection Control) and either negative control siRNA (siSCR) or PKC α siRNA (siPKC α). **(A)** Immunofluorescence (IF) was performed with an antibody against PKC α. Representative images are shown. Scale bar: 10 µm. **(B)** Based on the PKC α IF performed in (A), total PKC α signal was quantified for 173 siSCR control cells and 176 siPKC α cells based on three biological replicates. **(C)** HeLa cell lysates were subjected to Western blotting using antibodies against PKC α (top) and β-actin (bottom). The total integrated PKC α band intensity signal was quantified and normalized to the total protein signal measured by Ponceau staining. Each lane represents one biological replicate. The normalized total PKC α signal was 11161 ± 3724 for siSCR control and 5033 ± 2956 for siPKC α.
On scatter plots, dashed lines represent means. Unpaired, two-tailed t tests were performed. ****, $p<0.0001$.

Figure S4: Full-length western blots. Full-length western blots are shown for **(A)** Fig. S1C, **(B)** Fig. S2C, **(C)** Fig. S2F, and **(D)** Fig. S3C.

Figure S1:

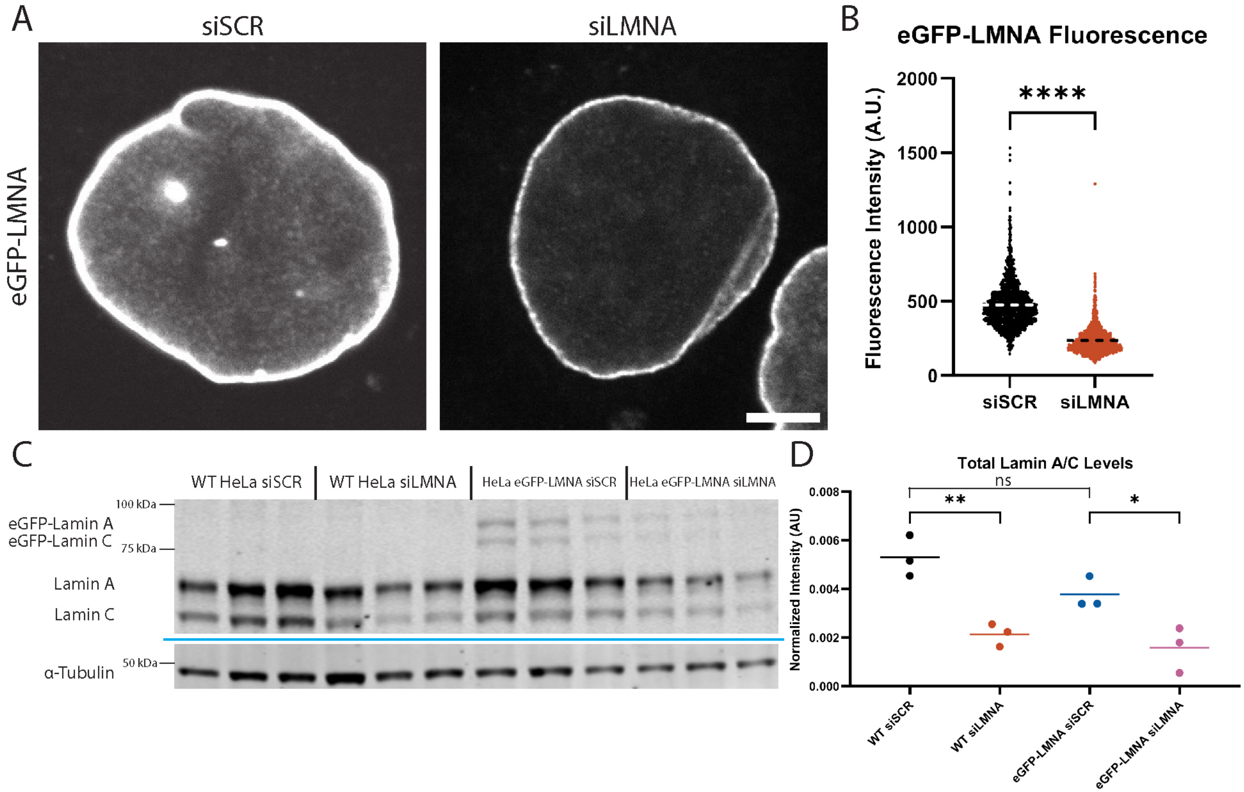
A
siSCR
siLMNA
eGFP-LMNA
B
eGFP-LMNA Fluorescence
Fluorescence Intensity (A.U.)
2000
1500
1000
500
0
****
siSCR
siLMNA
C
WT HeLa siSCR
WT HeLa siLMNA
HeLa eGFP-LMNA siSCR
HeLa eGFP-LMNA siLMNA
100 kDa
75 kDa
50 kDa
eGFP-Lamin A
eGFP-Lamin C
Lamin A
Lamin C
α-Tubulin
D
Total Lamin A/C Levels
Normalized Intensity (AU)
0.008
0.006
0.004
0.002
0.000
ns
**
*
WT siSCR
WT siLMNA
eGFP-LMNA siSCR
eGFP-LMNA siLMNA

Figure S2:

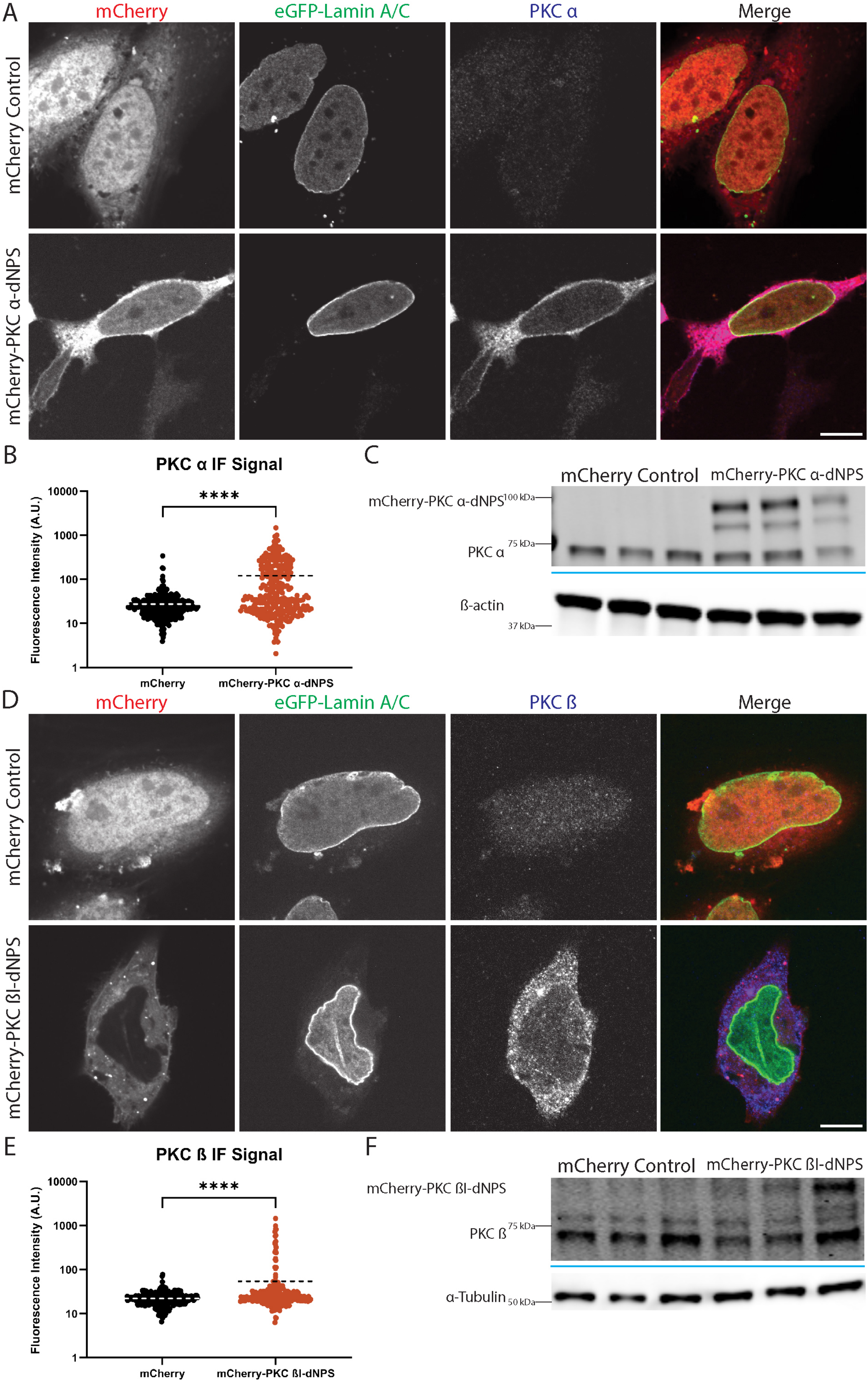
A
mCherry
eGFP-Lamin A/C
PKC α
Merge
mCherry Control
mCherry-PKC α-dNPS
B
PKC α IF Signal
****
Fluorescence Intensity (A.U.)
10000
1000
100
10
1
mCherry
mCherry-PKC α-dNPS
C
mCherry Control
mCherry-PKC α-dNPS
mCherry-PKC α-dNPS
100 kDa
75 kDa
PKC α
ß-actin
37 kDa
D
mCherry
eGFP-Lamin A/C
PKC ß
Merge
mCherry Control
mCherry-PKC ßI-dNPS
E
PKC ß IF Signal
****
Fluorescence Intensity (A.U.)
10000
1000
100
10
1
mCherry
mCherry-PKC ßI-dNPS
F
mCherry Control
mCherry-PKC ßI-dNPS
mCherry-PKC ßI-dNPS
75 kDa
PKC ß
α-Tubulin
50 kDa

Figure S3:

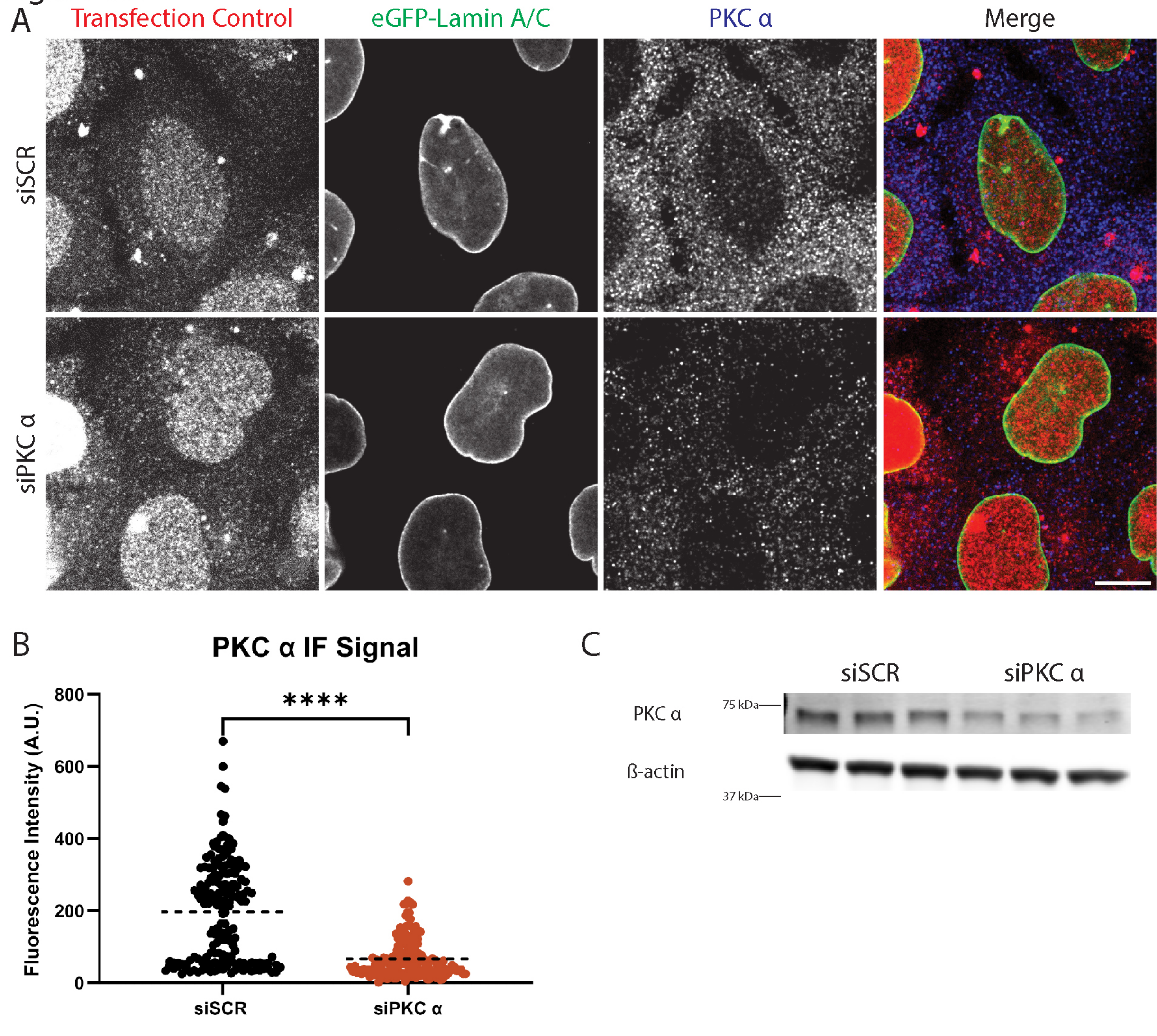
A
Transfection Control
eGFP-Lamin A/C
PKC α
Merge
siSCR
siPKC α
B
PKC α IF Signal
Fluorescence Intensity (A.U.)
800
600
400
200
0
****
siSCR
siPKC α
C
siSCR
siPKC α
PKC α
ß-actin
75 kDa
37 kDa

Figure S4:

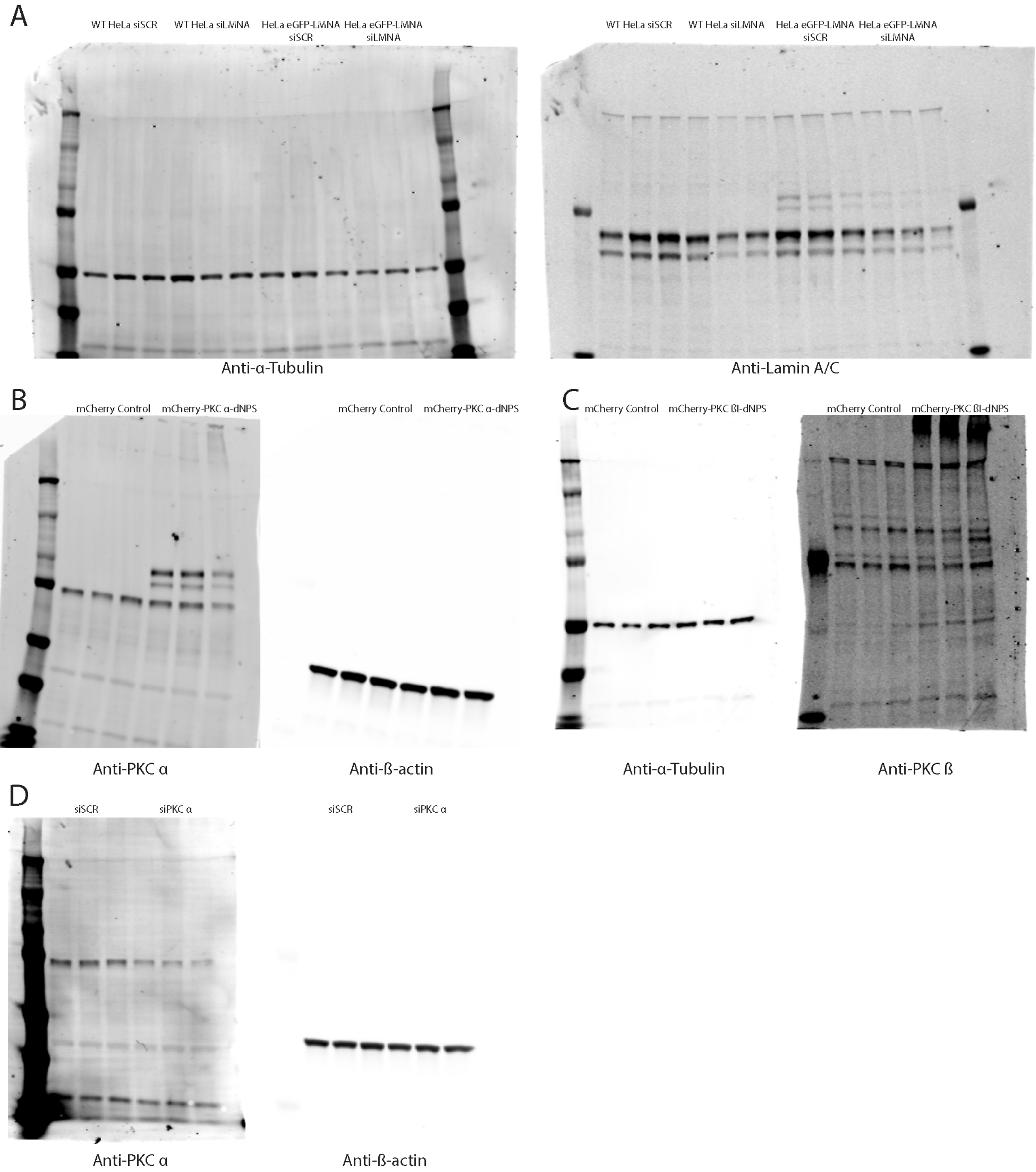
A
WT HeLa siSCR
WT HeLa siLMNA
HeLa eGFP-LMNA siSCR
HeLa eGFP-LMNA siLMNA
Anti-α-Tubulin
WT HeLa siSCR
WT HeLa siLMNA
HeLa eGFP-LMNA siSCR
HeLa eGFP-LMNA siLMNA
Anti-Lamin A/C
B
mCherry Control
mCherry-PKC α-dNPS
mCherry Control
mCherry-PKC α-dNPS
Anti-PKC α
Anti-ß-actin
C
mCherry Control
mCherry-PKC ßI-dNPS
mCherry Control
mCherry-PKC ßI-dNPS
Anti-α-Tubulin
Anti-PKC ß
D
siSCR
siPKC α
siSCR
siPKC α
Anti-PKC α
Anti-ß-actin
